# Supporting community annotation and user collaboration in the integrated microbial genomes (IMG) system

**DOI:** 10.1186/s12864-016-2629-y

**Published:** 2016-04-26

**Authors:** I-Min A. Chen, Victor M. Markowitz, Krishna Palaniappan, Ernest Szeto, Ken Chu, Jinghua Huang, Anna Ratner, Manoj Pillay, Michalis Hadjithomas, Marcel Huntemann, Natalia Mikhailova, Galina Ovchinnikova, Natalia N. Ivanova, Nikos C. Kyrpides

**Affiliations:** Biosciences Computing, Computational Research Division, Lawrence Berkeley, National Laboratory, Berkeley, California USA; Prokaryotic Super Program, DOE Joint Genome Institute, Walnut Creek, California USA

**Keywords:** Gene annotation, Functional curation, Manual curation, IMG, Metagenomics, Microbial genomics

## Abstract

**Background:**

The exponential growth of genomic data from next generation technologies renders traditional manual expert curation effort unsustainable. Many genomic systems have included community annotation tools to address the problem. Most of these systems adopted a “Wiki-based” approach to take advantage of existing wiki technologies, but encountered obstacles in issues such as usability, authorship recognition, information reliability and incentive for community participation.

**Results:**

Here, we present a different approach, relying on tightly integrated method rather than “Wiki-based” method, to support community annotation and user collaboration in the Integrated Microbial Genomes (IMG) system. The IMG approach allows users to use existing IMG data warehouse and analysis tools to add gene, pathway and biosynthetic cluster annotations, to analyze/reorganize contigs, genes and functions using workspace datasets, and to share private user annotations and workspace datasets with collaborators. We show that the annotation effort using IMG can be part of the research process to overcome the user incentive and authorship recognition problems thus fostering collaboration among domain experts. The usability and reliability issues are addressed by the integration of curated information and analysis tools in IMG, together with DOE Joint Genome Institute (JGI) expert review.

**Conclusion:**

By incorporating annotation operations into IMG, we provide an integrated environment for users to perform deeper and extended data analysis and annotation in a single system that can lead to publications and community knowledge sharing as shown in the case studies.

## Background

Traditional genomic annotation relies heavily on manual expert curation [1, 2]. With the arrival of next generation technologies, genomic data grows exponentially [3] while expert curation increasingly lags behind [4]. Many systems have been developed to facilitate community-based curation to address this problem. Most of these systems are “wiki-based”; e.g., Gene Wiki [5] and WikiGenes [6] for gene annotations, RNA WikiProject [7] and miRBase [8] for RNA annotations, WikiProteins [9] and TOPSAN [10] for protein annotations, and WikiPathways [11] for pathway annotations. In general, these systems use wiki technologies to create a wiki page or “stub” for each gene/protein/etc., remove potential duplicates, create hyperlinks to relevant information, and let registered users be contributors to add community annotations. Even though wiki technologies enable community annotations, there are additional issues to be resolved:

- Authorship matters in scientific annotations, because scientists need recognition, and the scientific community needs additional information to judge the authority of annotations [6, 10].
- Even though Wikipedia is considered reliable in general, it is not subject to strict peer review. Without proper quality control, there can be a large number of dubious annotations [5, 8, 10].
- Some wiki pages will need to remain private in a short period of time before they are ready for publication [11].
- The usability issue needs to be addressed to encourage community participation [11].
- The level of community participation is not high due to lack of incentive [4].
- Some areas require special expertise and therefore are not suitable for annotations by community at large [7].

In order to address the above issues, various systems introduced mechanisms to track authorship, to limit editing to registered expert users, to incorporate expert review and validation, and to improve usability [4, 5, 7, 8, 10, 11]. Proposals have also been made to provide incentive and recognition of authorship [4, 5].

There are also non-wiki based systems such as ORegAnno [12] and BioGPS [13], which are standalone curation systems implemented using database technology and web-based user interface. Such systems avoid some problems of the wiki-based systems; however, they require more development efforts to implement data storage and user interface, and still need to address issues such as information validation, usability and community participation even though integrated genomic information provided by such systems helps improving the usability, and registered users provide the base for community participation.

The exponential data growth problem has also been encountered in the Integrated Microbial Genome System (IMG). As of January 2016, IMG has more than 38,000 archaeal, bacterial and eukaryotic genomes, with more than 140 million genes in those genomes. Many genes are simply annotated as hypothetic proteins without more specific information. There is also an urgent need to support community annotation and user collaboration in IMG. Therefore, MyIMG annotation, which is a tool tightly integrated into IMG and is not wiki based, has been developed.

There are several reasons we follow the integrated system approach rather than the wiki-based approach to support annotation and collaboration. First, wiki-based approaches require identifying and removing duplications so that there won’t be redundant and confusing web pages. All the above mentioned wiki-based systems only have thousands or tens of thousands of objects (genes, proteins, etc.), and it is not too difficult to identify duplications for removal. In contrast, IMG has more than 140 M isolate genes, and it is not trivial to identify non-redundant genes. Continuous data loading and marking as obsolete, older and redundant versions of genomes in IMG further complicate the problem. Second, IMG already provides many comparative analysis tools to aid users in finding additional information or locating potential “missing” genes that were overlooked by the gene calling pipelines. That is, IMG not only offers a place for users to add and share annotations but also provides tools to help users performing annotations. In addition, IMG provides tools for users to save genomes, genes, scaffolds and functions into Workspace datasets, and users can also use these Workspace functions to reorganize various objects (e.g., genes, functions) to suit their research needs. By incorporating annotation operations into IMG, we provide an integrated environment for users to perform extended data analysis and annotation in a single system that can lead to publications and community knowledge sharing as illustrated in the rest of this paper.

The integrated system approach does not automatically resolve all problems encountered by the wiki-based approach. We still need to address issues such as usability, authorship recognition and tracing, user incentive, and reliability even though existing IMG features already provide at least partial solutions. Moreover, IMG has more than 14,000 registered users from 93 countries as of January 2016, which provides a solid base for community participation. Involvement of DOE Joint Genome Institute (JGI) experts also helps with the reliability and certain usability issues.

## Implementation

### IMG system

IMG UI (http://img.jgi.doe.gov/mer) is a free web based tool, open to all scientists worldwide for the annotation, analysis, and distribution of their own genome and metagenome datasets. The IMG web UI works on all modern computers on the following operation systems: Win 7/10, Mac OS and Linux. IMG’s recommend web browsers are Google’s Chrome version 49+ and FireFox version 45+ with JavaScript and Cookies enabled. Also, a high speed internet connection with a minimum of 5 Mbps is required to view IMG’s large data sets. IMG uses some third parties tools like Artemis which requires Java version 1.8+ to be installed on the user’s computer. All other IMG’s analysis tools use open source bioinformatics software: BLAST, Mummer, EMBOSS, Bioperl, which run on IMG’s Linux servers. Where the IMG’s Perl web framework creates viewers using YUI JavaScript and D3 JavaScript libraries, to create tables, charts and graphs.

IMG data warehouse is a hybrid system consisting of Oracle 11 g databases, SQLite databases and file systems. Accesses to the data warehouse are through IMG UI.

More information regarding the IMG system can be found at: http://img.jgi.doe.gov/.

### IMG user groups

Biologists analyzing genomic sequences usually work in groups. They wish to share private genomic annotations with collaborators until the research results are accepted for publication. After that, the annotations can be available for general public consumption. Each scientist can participate in multiple research groups, and a group can be consisting of colleagues of the same or interdisciplinary fields or mentors and students. This effectively captures all four collaboration types: peer-to-peer, mentor-student, interdisciplinary, and producer-consumer as described in [14].

IMG started supporting user groups in 2007. Groups were created for JGI internal research groups, university professor and student groups, collaborative annotation jamborees, etc. Initially groups were created through email requests, and each user could belong to only one group. Recent extension allows users to create their own groups using IMG’s User Interface, and a user can belong to multiple groups. There are currently more than 70 user groups (as of January 2016).

New features allow users to actively manage their groups and to share information for collaboration. There are 3 possible roles for a user group: (1) owner, (2) co-owner, who also has the administrative privilege, and (3) member. Owners and co-owners can update group description, add members to a group, or remove members from a group. Even though members cannot add or remove other members, they can decide to withdraw from a group.

All group members can post news to share with other group members. News can include notifications, new publications, links to shared documentation (e.g., Google Doc) for collaboration, etc. Members can also grant access permission of their private genomes to other group members. Group members can also share annotations and workspace data sets (to be described below).

### IMG gene annotations

We have started supporting IMG gene annotations since IMG ER 2.0 [15]; new features are continuously being added. There are two types of gene annotations:

- MyIMG gene annotation allows users to add additional information to existing genes or to make the genes obsolete. Each MyIMG gene annotation includes the following fields that can be manually edited: gene product name, gene symbol, description, enzyme EC number, Pubmed ID, notes, and whether the gene is marked deleted.
- Missing gene annotation allows users to add new genes that have been missed by gene calling pipelines. Each missing gene annotation includes the following fields: gene product name, gene symbol, locus type (protein coding gene, tRNA, rRNA, etc.), locus tag, coordinates on a scaffold, strand, and enzyme EC number.

Tools for finding candidate gene product name using function comparison, finding missing enzymes using KEGG pathways, and finding missing genes using Phylogenetic Profiler have already been described [15]. Since then new tools are constantly being added. Here, we provide comprehensive gene annotation methods using both existing and recently developed tools.

#### Sequence similarity based annotation

**Fig. 1:**
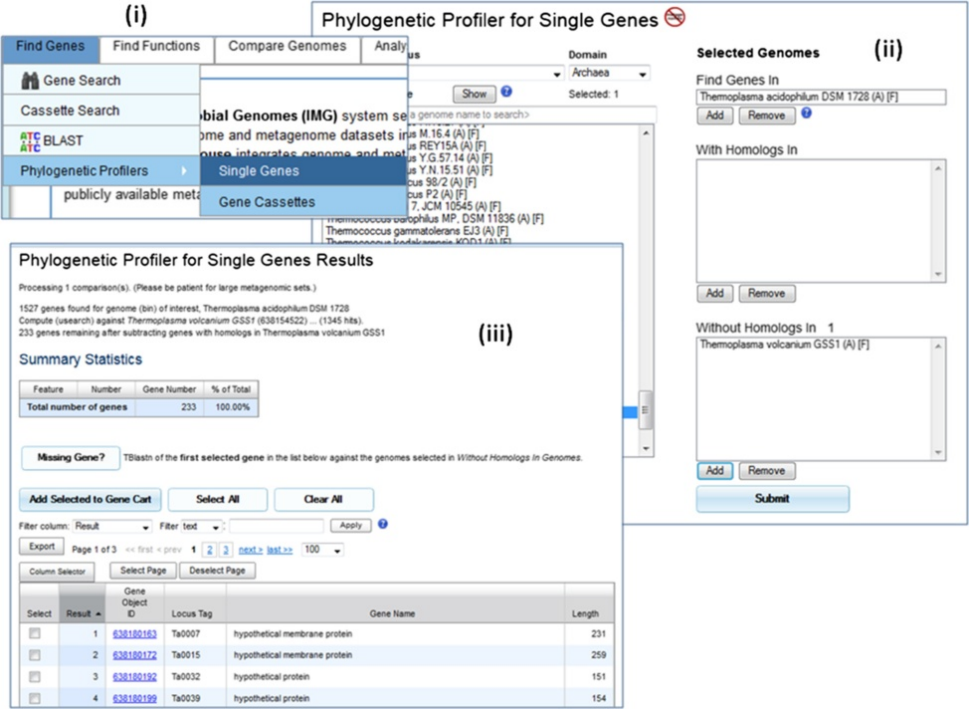
Using Phylogenetic Profiler to Find Gene Annotations and Missing Genes. From the Find Genes menu item, a user can select Phylogenetic Profilers: Single Genes submenu (Fig. 1 (**i**)) to start investigating genes in a selected candidate genome with or without homologs in other closely related genomes (Fig. 1 (**ii**)). While the “With Homologs” option is useful for additional MyIMG gene annotations, the “Without Homologs” option provides a list of potential missing genes for further investigation (Fig. 1 (**iii**)). To investigate a potential missing gene, the user first selects the gene and then clicks on the “Missing Gene?” button (Fig. 1 (**iii**)). Potential missing genes identified by TBlastn search will be displayed

The most common way to acquire additional gene information is by using sequence similarity search such as BLAST. If a gene *g1* is found to be matching a better annotated gene *g2*, information of *g2* such as gene product name, gene symbol, enzyme and protein information can be transferred to *g1*. Since it is very time consuming to check each gene of a genome using BLAST, IMG provides an analysis tool for massive gene comparison using Phylogenetic Profiler as shown in Fig. 1 [15].

Phylogenetic Profiler allows users to find genes of a target genome with or without homologs in one or more closely related reference genomes. For those genes with homologs, functional annotation of homolog genes can be transferred to the genes. Potential missing genes can be identified for genes in reference genomes that do not have homologs in the target genome. After users identify potential missing genes, they can then go to MyIMG to add missing gene annotations of those genes. Similar to MyIMG annotations, missing gene information is private by default and can be shared among group members.

Sequence similarity based approach, though simple, has its limitations. This approach relies on the availability of closely related reference genomes with better annotations. In addition, even though Phylogenetic Profile provides a list of potential missing genes for investigation, it is still tedious and time consuming to go through the list. For better results, sequence similarity based approach can be combined with additional approaches to be described below.

#### Function based annotation

Users can annotate a gene with more meaningful name (i.e., other than “hypothetical protein”) simply by checking functional annotation of the same gene. For example, genes without a product name but with evidence of potential functional annotation or with product name but without any evidence of functional annotation are candidates for product name review and curation [15].

Function Profile is a widely used tool to check whether a set of functions is present in closely related genomes. Users can take advantage of various function categories in IMG to help gathering a set of functions for running profile. For example,

- COG Category: A COG category consists of a set of COG functions [16].
- Pfam Clan: A Pfam clan consists of a set of Pfam functions [17].
- TIGRfam Role: A TIGRfam role consists of a set of TIGRfam functions [18].
- KEGG Pathway: A KEGG pathway consists of a set of enzymes [19].
- KEGG Module: A KEGG module consists of a set of KO terms [19].
- IMG Pathway: An IMG pathway consists of a set of ordered reactions; each reaction is linked to one or more IMG terms [20].
- IMG Parts List: An IMG parts list consists of a set of IMG terms of related function.

**Fig. 2:**
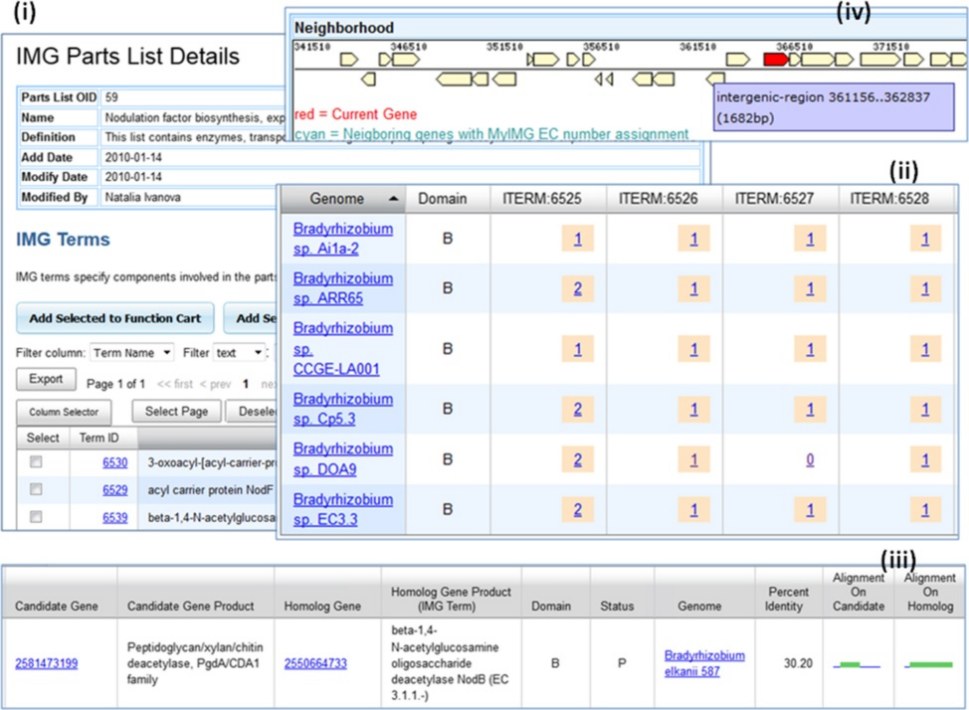
Finding Missing IMG Terms Using Function Profile. A user first selects an IMG Part List *Nodulation factor biosynthesis, export and regulation* to load all component IMG terms into Function Cart (Fig. 2 (**i**)). All *Bradyrhizobium* genomes are supposed to have genes associated with these terms. However, some terms are missing in certain genomes (Fig. 2 (**ii**)). Clicking on the zero count will lead to searching potential genes using BLAST as the result shown in Fig. 2 (**iii**). Since microbial genes with related functions tend to be close together on the scaffold, an alternative approach is to investigate intergenic regions of genes with functions to look for potential missing genes (Fig. 2 (**iv**))

Figure 2 shows an example of using IMG Part List *Nodulation factor biosynthesis, export and regulation*, which contains 22 IMG terms for enzymes, transporters and regulators participating in biosynthesis and export of nodulation factors, to find missing IMG terms in *Bradyrhizobium* genomes.

IMG also provides tools for users to investigate possible missing enzymes based on KEGG pathways as shown in [15]. The tool uses both sequence similarity search and pre-computed gene-KO (KEGG Orthology) information in the database, which includes a list of genes not being annotated with enzymes because the association did not make the strict cutoff determined by the IMG data processing pipeline. Users can review the list and decide whether to add MyIMG gene-enzyme annotations using their professional judgment.

**Fig. 3:**
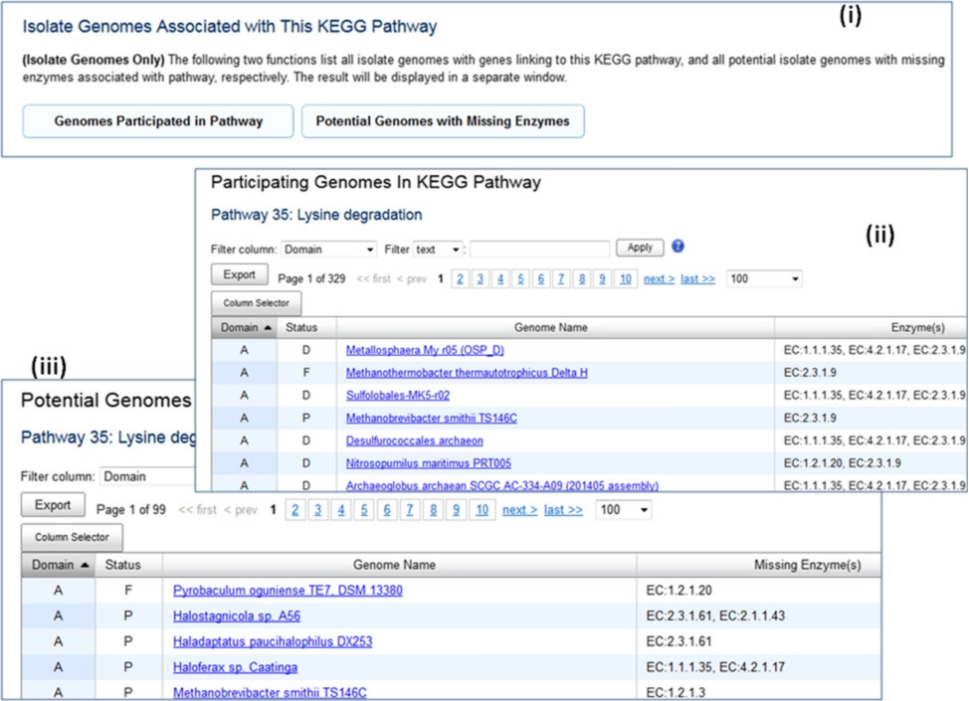
List of participating genomes and potential genomes with Missing Enzymes. Two new functions are provided to help users to narrow down genome searches (Fig. 3 (**i**)). Participating Genomes in KEGG Pathway gives users a list of all genomes participated in the selected pathway together with the enzymes (Fig. 3 (**ii**)). Potential Genomes With Missing Enzymes function gives users a list of potential genomes with missing enzymes to investigate (Fig. 3 (**iii**))

Even though the finding missing enzyme function has been introduced since 2009, it has not been widely used. We realize that with more than 38,000 archaeal, bacterial and eukaryotic genomes and 474 KEGG pathways in IMG, trying to find missing enzymes using the above tool is like finding needle in haystack. Therefore, we recently added additional functions (at the bottom of View Map for Selected Genomes page) to show all genomes participated in the selected KEGG pathway, and potential genomes with missing enzymes to help narrowing down candidate genomes (see Fig. 3).

**Fig. 4:**
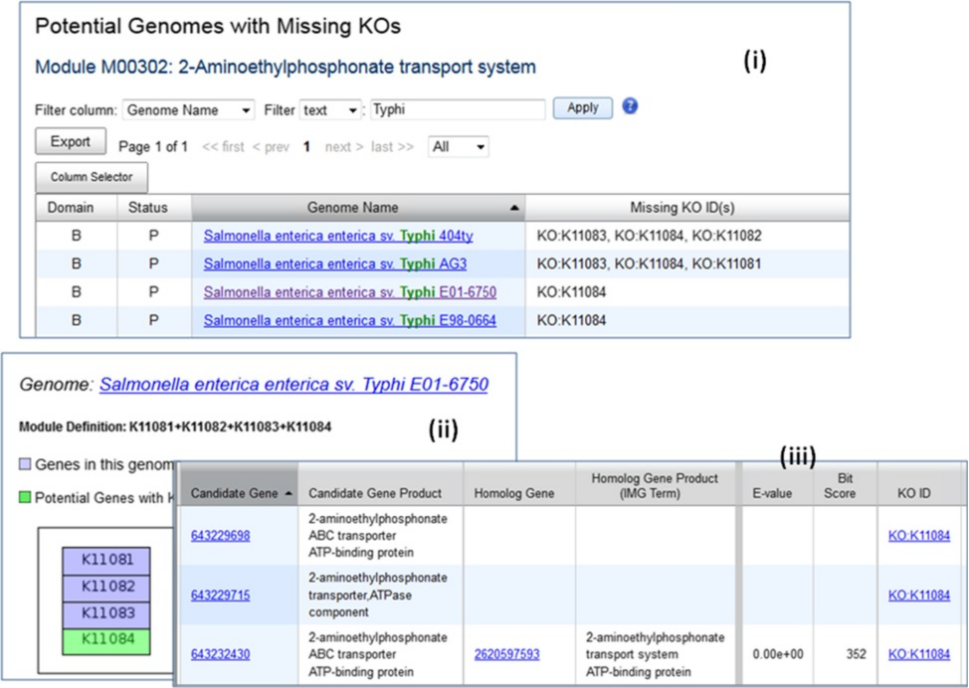
Finding Genes with Missing KO Terms. Many *Salmonella enterica* genomes have complete KO Module M00302 *2-Aminoethylphosphonate transport system*. While *Salmonella enterica enterica sv. Typhi E01-6750* is shown to be missing a KO Term K11084 (Fig. 4 (**i**)). When a user displays KEGG Module Map of M00302, he/she can clearly see that the genome has genes associated with 3 other KO terms but not K11084 (Fig. 4 (**ii**)). By clicking on the “green” KO term on the map, the user can use a new IMG tools to identify 3 genes that can potentially be associated with this KO term

For many researchers, KEGG pathways are often too broad, and they’d rather rely on KEGG modules with more restricted focus. Therefore, we recently introduced colored KEGG module maps and finding missing functions using KEGG modules similar to what we have done for KEGG pathways. An example of finding genes missing KO terms is shown in Fig. 4.

IMG phenotype prediction and pathway assertion also provides a way for users to identify genes missing IMG term assignment. It is shown in [21] that *Burkholderia sp. SJ98* contains genes for chorismate synthesis. However, the genome does not have IMG Pathway 146 *Chorismate synthesis* asserted. The pathway assertion status is unknown due to missing IMG term 335 *shikimate dehydrogenase (EC 1.1.1.25)* even though there are ortholog genes annotated with this term. After using sequence similarity search, 2 genes were found to be potential candidates of missing term assignment.

**Fig. 5:**
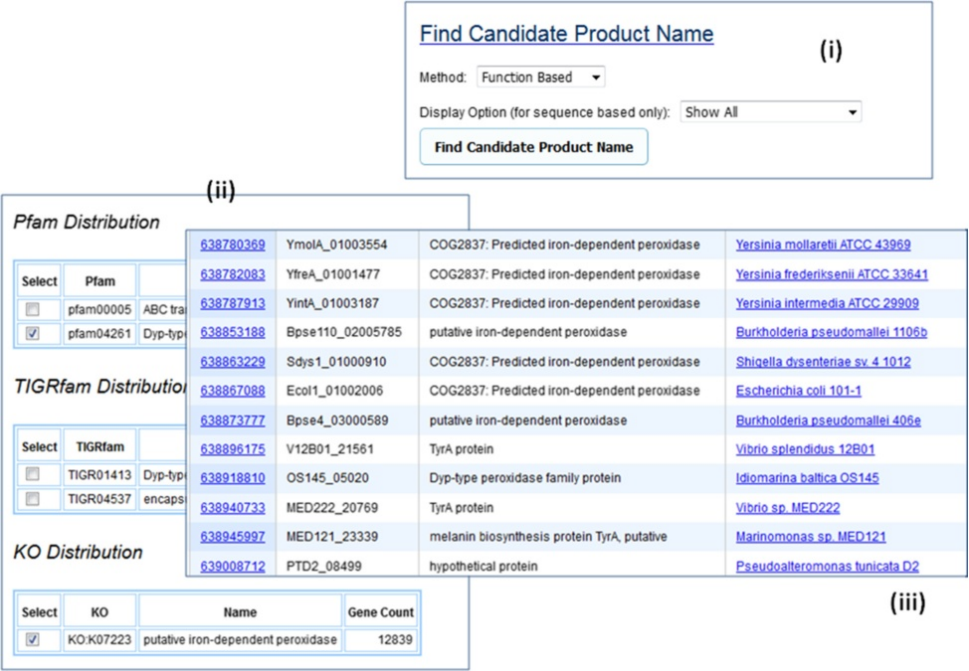
Using Function Based Production Name Method to aid MyIMG annotation. A gene may be assigned with a product name “hypothetic protein” due to lack of information even though it is association with some functional assignment. Using the Function Based finding candidate product name method from the Gene Detail page (Fig. 5 (**i**)), users will be able to see the function distribution of other public genes with the same functional assignment (Fig. 5 (**ii**)). The List Genes function shows all public genes with selected functional assignment (Fig. 5 (**iii**)), which can provide hint for MyIMG annotation of the candidate gene

Another new tool in the gene detail page allows users to find the function distribution of other public genes in IMG with the same functional association of a particular gene. Users can then view those public genes with selected functional assignment to find a more meaningful name of the candidate gene (see Fig. 5).

#### Gene neighborhood based annotation

Gene neighborhood is another common tool used for gene annotations. Simply by looking at the gene neighborhood diagram, a user can sometimes tell whether a gene is too long or too short, and whether there are overlapping genes. Long intergenic region or presence of genes in reference genomes shown in the gene neighborhood can also suggest the existence of missing genes. Expert users often rely on sequence visualization and analysis tools such as Artemis [22] to identify missing genes.

**Fig. 6:**
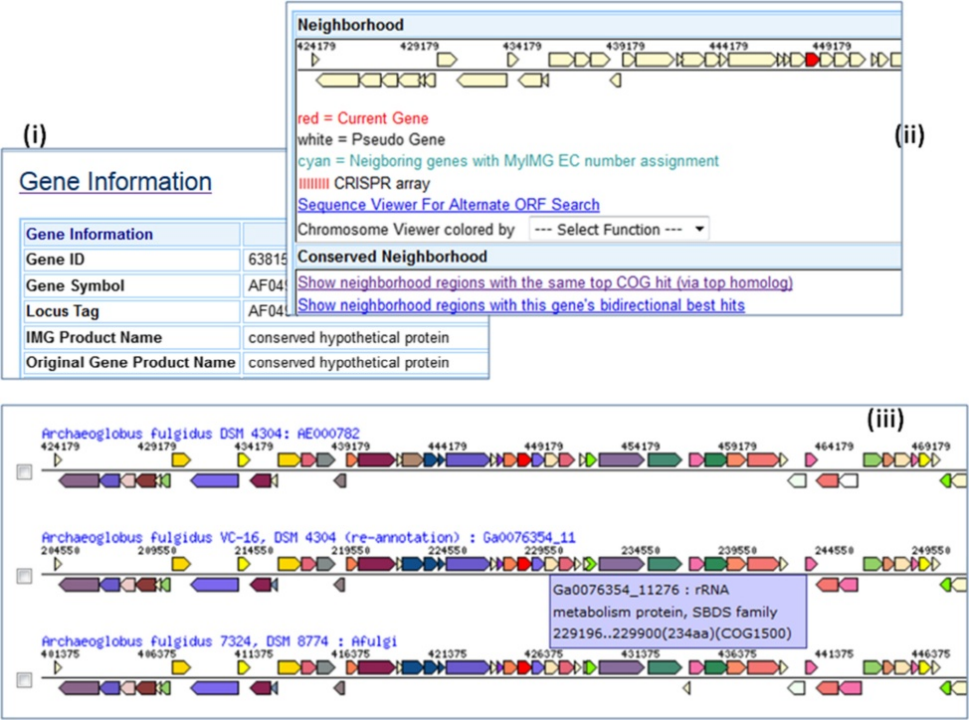
Using Gene Neighborhood to aid MyIMG annotation. A gene may be assigned with a product name “conserved hypothetic protein” due to lack of information (Fig. 6 (**i**)). However, from the gene neighborhood with the same top COG hit (Fig. 6 (**ii**)) shows that there are other similar genes with more meaningful product names (Fig. 5 (**iii**)). In this case, a user can add MyIMG annotation product name such as “putative RNA-associated protein”

An example of using gene neighborhood to aid MyIMG gene annotation is shown in Fig. 6.

Since microbial genes of related functions tend to locate closely together on a scaffold, gene neighborhood method can be combined with function based method to find missing genes. Following the nodulation factor example in Fig. 2, when a genome is missing a function as shown in Function Profile, there can be two possibilities: (i) one or more genes of the genome should have been annotated with this function, or (ii) the gene calling pipeline missed calling gene(s) for the function. In case (ii) a user can investigate intergenic regions of genes with functions to spot potential missing genes (Fig. 2(iv)).

Spurious genes can be added by incorrect gene calling programs. When gene neighborhood with the same top COG hit returns no result, there is a possibility that the gene may not be real. When gene neighborhood shows overlapping genes, it is also a good indication that one or more genes are incorrectly called. Domain experts can also identify erroneous genes by checking the sequence data. Spurious genes can be genes that are too long, too short, with incorrect starting codon, etc [23]. IMG users can mark deleting genes by creating MyIMG gene annotations with “Remove Gene from Genome?” field set to Yes.

The review of genes and their functional annotations may lead to the identification of missing genes. For every marked deleted gene, it is possible to identify one or more genes in the neighborhood. For example,

- Gene too short: There may be a longer gene.
- Gene too long: There may be one or more shorter genes.
- Incorrect starting codon: There can be a real gene downstream or upstream.

#### Sharing and comparing MyIMG annotations

If a user belongs to one or more IMG groups, then the user can view all MyIMG annotations by group members with the following restrictions:

- The user must have access permission to the genomes. All MyIMG annotations on private genomes will only be visible to other group members that have access permission to the private genomes. Shared or public MyIMG annotations on public genomes are not restricted.
- Those MyIMG annotations must be either public or shared by the authors of the annotations. (An author can selectively share MyIMG annotations with different groups; e.g., sharing with Group 1, but not with Group 2. In this case, Group 1 members can view the MyIMG annotations, but Group 2 members cannot.)
- The user can only view, but not modify, MyIMG annotations by other group members.

The “View Group Annotations” option in MyIMG allows a user to view shared annotations by group members. Many IMG users have used this feature for group annotations with colleagues. For users who belong to multiple IMG groups, there will be a dropdown selection for users to switch groups.

A recently introduced “Update Group Sharing” section allows users to change the group sharing option of selected annotations. There are two options for a user to share his/her MyIMG annotations:

1. sharing all MyIMG annotations of selected genome(s);
2. sharing individually selected MyIMG annotations.

MyIMG annotations are private by default. However, IMG users can release any of their MyIMG annotations to public. Public MyIMG annotations are visible to all IMG users provided that users have access permissions to the corresponding genomes. Public MyIMG annotations on public genomes can be viewed by all users. Moreover, since IMG ER is an “Expert Review” site, all public missing gene annotations can be reviewed and modified by JGI experts.

The “Show All User Annotation” function in a Gene Detail page allows a user to view all MyIMG annotations available to him/her on this particular gene. All gene annotations together with curator names are listed in a table for easy comparison.

### Pathway annotations

**Fig. 7:**
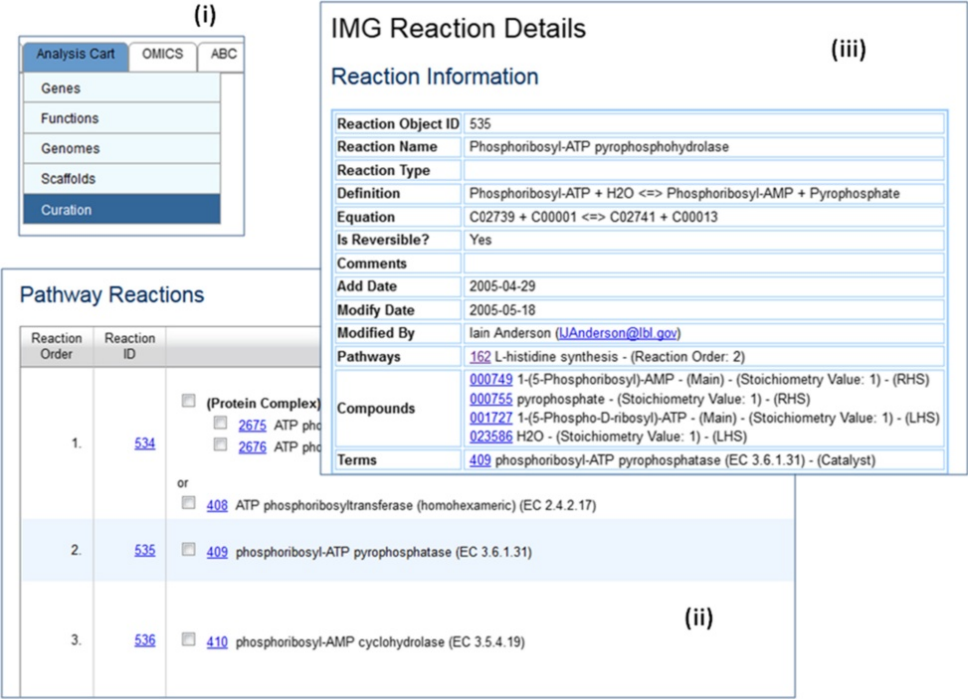
IMG Pathway Curation. Users with curation privilege will be able to see an additional Curation submenu item in the Analysis Cart (Fig. 7 (**i**)). An IMG Pathway is consist of one or more sequential, alternative, and/or optional reactions (Fig. 7 (**ii**)), while each reaction is consist of definition, equation, compounds as reactant, product or catalyst and related IMG terms (Fig. 7 (**iii**))

Some research areas require profound domain knowledge and are best left to only expert annotations. Therefore, many systems have restricted certain editing capabilities to experts only [7]. Poor annotations can lead to multiple dubious entries [8]. This is especially true in IMG where pathway assertion results are used to predict phenotypes [24]. Hence, only JGI experts and a few external users with special permissions are allowed to IMG pathway curation [20] (Fig. 7). (Interested users can contact us to request for the pathway curation privilege.)

Due to the tremendous effort required to define a pathway, IMG currently has only 900+ IMG pathways in the database. All IMG pathways are public to all users.

### Biosynthetic cluster annotations

Biosynthetic clusters and secondary metabolites (or natural products) are recent additions to the IMG system [25]. There is currently increasing research interest in biosynthetic clusters and natural products. However, the amount of experimentally available data in this area is scarce. Among more than 1 million experimentally verified and predicted biosynthetic clusters in IMG, only less than 0.2 % of the clusters are associated with any secondary metabolites.

**Fig. 8:**
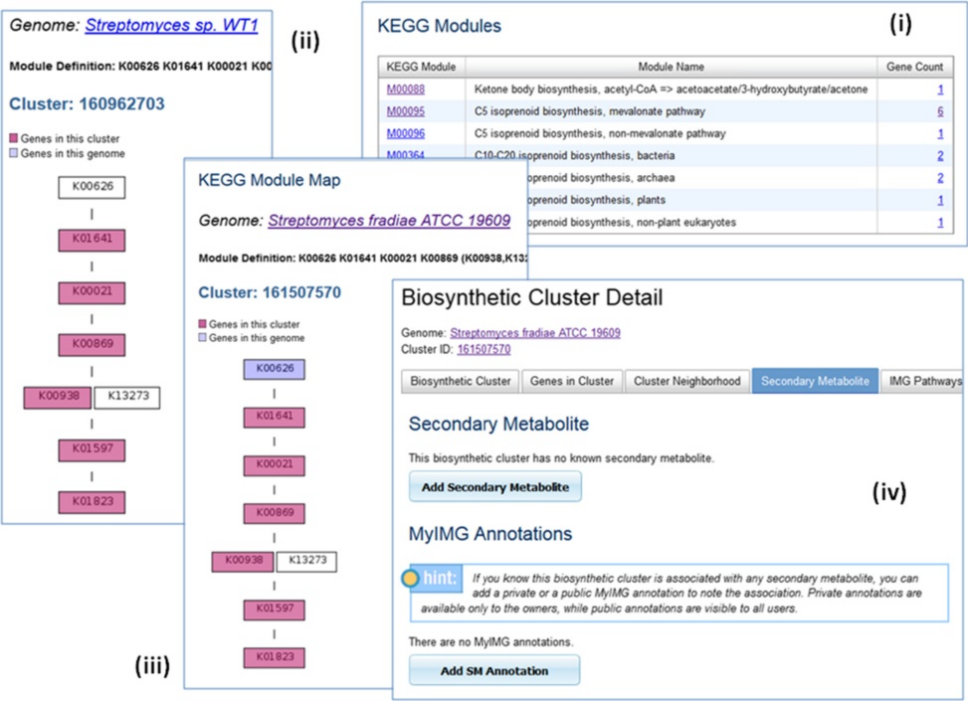
Biosynthetic Cluster and Secondary Metabolite Annotation. Experimentally verified biosynthetic clusters are associated with secondary metabolites, while such information is missing from predicted biosynthetic clusters. Biosynthetic cluster 160962703 of *Streptomyces sp. WT1* is an experimentally verified cluster (Genbank Accession JN207130) associated with natural product Mevalonate. Genes of this cluster participate in 7 KEGG modules (Fig. 8 (**i**)). KEGG Module Map M00095 *C5 isoprenoid biosynthesis, mevalonate pathway* for this cluster shows that genes in this cluster are linked to 6 of the KO terms (Fig. 8 (**ii**)). Predicted biosynthetic cluster 161507570 of *Streptomyces fradiae ATCC 19609* does not have any secondary metabolite information. However, it contains 6 genes associated with the same 6 KO terms of M00095, which is a good indication that the cluster can produce the same secondary metabolite (Fig. 8 (**iii**)). Users can use the “Add SM Annotation” function to annotate the association (Fig. 8 (**iv**))

It is possible to associate biosynthetic clusters to secondary metabolites using sequence similarity search and pathway structures as described in a case study in [25]. Users can also use additional pathway or KEGG module information to discover or predict secondary metabolites as shown in an example in Fig. 8.

Users can use the “Add SM Annotation” function to annotate their discovery. Each MyIMG SM annotation includes a compound selection, NCBI accession and taxon information (if any) and free text comments. The annotation will remain private until the owner decides to release the information to community at large. We are hoping that with the introduction of IMG-ABC and the new MyIMG SM annotation features, more community users will collaborate to contribute to the advance in this research area.

### Workspace for annotation and collaboration

IMG Workspace allows users to store their work in progress in four types of datasets: genome sets, scaffold sets, gene sets and function sets. Unlike analysis carts, which are transient and are deleted after each session, workspace datasets are stored permanently until users explicitly delete them. It can be considered as a form of electronic notebooks for genomic data.

Besides data storage function, workspace can also be used for additional user annotation and collaboration, which cannot be achieved by using MyIMG annotations alone. We will describe these additional functions immediately below.

#### Workspace scaffold sets for organizing new genomes or metagenomes

Metagenome “binning” involves isolating certain scaffolds from a metagenomic dataset in order to remove contamination or to extract isolate genomes or single cells from the metagenome [26]. Many IMG analysis tools such as Phylogenetic Distribution, Kmer Frequency, Function Profile have been widely used for metagenome binning. Isolated or de-contaminated scaffolds can be saved into workspace scaffold sets, which can be further investigated using additional analysis tools provided by IMG. Users can also export the nucleotide sequence of scaffolds in a particular dataset to resubmit to IMG as a new genome or metagenome.

#### Workspace gene sets for creating new biosynthetic clusters

**Fig. 9:**
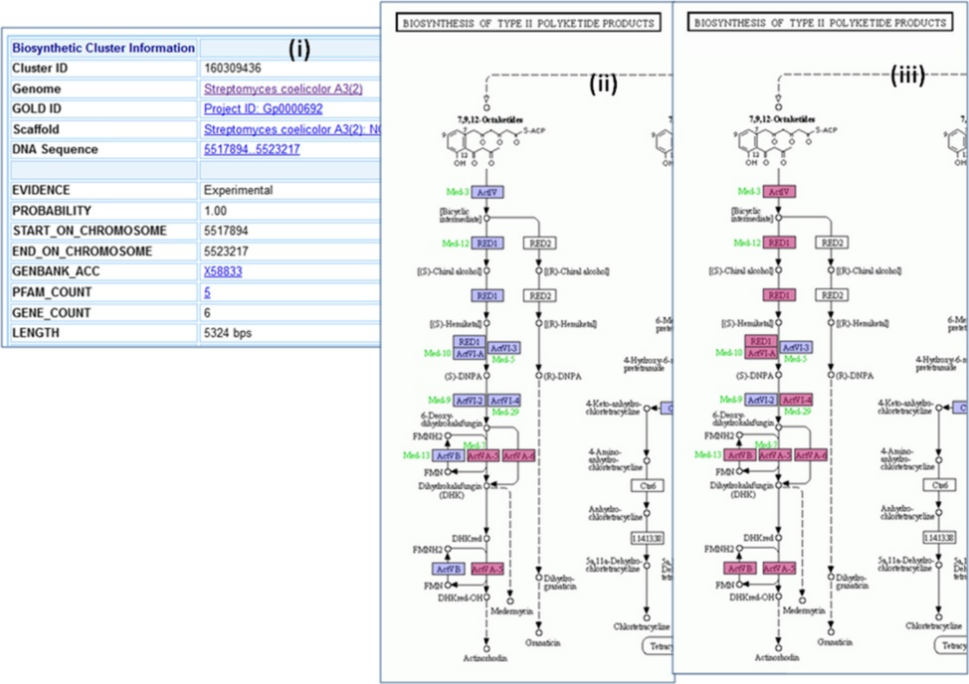
KEGG Map Display of Biosynthetic Cluster Genes. An experimentally verified biosynthetic cluster from NCBI with Genbank ID X58833 has 6 genes (Fig. 9 (**i**)). The KEGG Map shows the genes in this cluster only partially covers the Actinorhodin pathway. The boxes colored in magenta in the pathway map are linked to genes of this cluster, while the boxes colored in purple are genes in the same genome but not in the cluster (Fig. 9 (**ii**)). By adding 5 additional upstream and downstream genes, a new cluster will be able to cover the entire pathway (Fig. 9 (**iii**))

IMG-ABC system [25] includes more than 1 million experimentally verified and predicted biosynthetic clusters. In each biosynthetic cluster detail page, there are additional information showing secondary metabolites associated with the cluster and pathway participation of genes in the cluster. KEGG Map display of a biosynthetic cluster shows not only genes of this cluster but also other genes in the genome not in the cluster. In this way, a user can see clearly how well a cluster covers a pathway. An example in Fig. 9 shows a biosynthetic cluster that covers only portion of a pathway, while a new cluster with additional genes upstream and downstream will be able to cover an entire path.

Workspace gene set can be used as a tool for users to annotate their own biosynthetic clusters. A user can start with loading genes of an experimentally verified or predicted biosynthetic cluster into Gene Cart. In addition to biosynthetic clusters that are already in IMG, it is also possible for users to find genes mentioned in literature but not included in any IMG gene clusters. Additional genes on the same scaffold can be added based on analysis results from various IMG tools such as the KEGG Map display example described above. It is also possible to detect genes that should have been excluded. The final analysis result can be saved as a workspace gene set with a meaningful name. A Genbank-format file can be generated to include all genes in the final result, and the file can be submitted to IMG as a new genome fragment. Alternatively, a user can also obtain a portion of the scaffold based on gene coordinates and then submit the sequence to IMG as a new genome fragment.

#### Workspace function sets for defining pathways

**Fig. 10:**
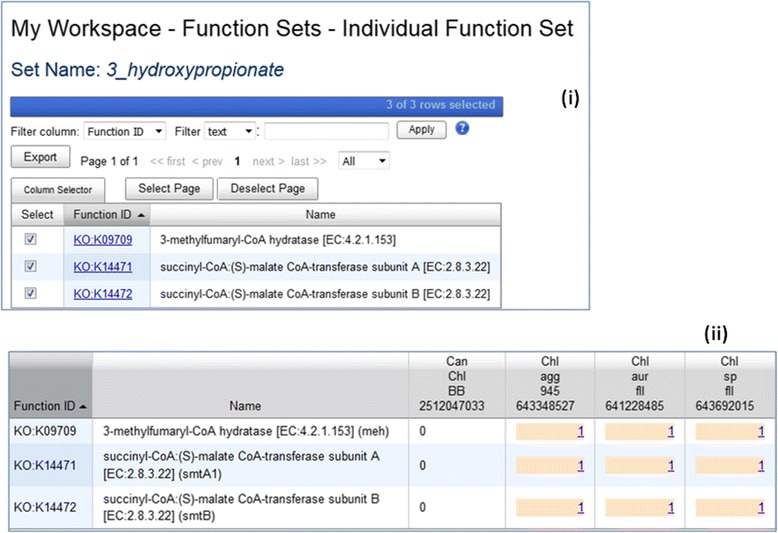
Make your own pathway and check assertion. A user can create a new “pathway” by adding functions into a workspace function set. For example, a user can create a “3 hydroxypropionate” pathway to include 3 KO terms K09709, K14471 and K14472 (Fig. 10 (**i**)). Function-Genome Profile then shows which genomes are “asserted” for this new pathway (Fig. 10 (**ii**))

For IMG users who are not interested in active pathway curation or who do not have the curation permission, it is still possible for them to “make their own pathway” using workspace function sets. A user can start with studying an IMG pathway, a MetaCyc pathway, a KEGG pathway or a KEGG Module to collect all or some of the functions (IMG terms, KO terms or enzymes). The user can then check pathway assertion of various genomes using the Genome Set Function Profile in Workspace or using the Function-Genome Profile provided in Analysis Cart (see Fig. 10). This tool enables users to try out pathway construction that is not limited to a single type of functions (e.g., IMG terms or enzymes only) and contributes to future pathway curation.

#### Shared workspace for collaboration

Ever since the introduction of Workspace to the IMG system in 2011, more than 8.7 % of all IMG registered users have used Workspace to store more than 77,000 datasets for their analysis (as of January 2016). We have described how IMG users can use workspace scaffold sets or gene sets to perform “above gene level” annotations to form new genomes or biosynthetic clusters, and use workspace function sets to make their own pathways. To perform group annotation, users can share their workspace datasets with their colleagues. Previously workspace dataset sharing was achieved through exporting and importing datasets, which is not only cumbersome but also does not support interactive analysis. We have recently introduced dynamic workspace dataset sharing within IMG groups to overcome the obstacle.

If a user belongs to one or more IMG groups, then the dataset list in Workspace will have 2 additional columns:

- Owner: the owner of a dataset (either “me” or name of a group member);
- Shared with Group: IMG group(s) having access to this dataset.

All Workspace datasets including genome, gene, scaffold and function can be shared. Users not only can view the content of a shared dataset but also can use shared datasets to perform analysis. However, users do not have editing privilege of others’ datasets; that is, they cannot edit or delete a dataset that they do not own. Alternatively, a user can copy a shared dataset content and then edit the new version of his/her own.

With the introduction of workspace dataset sharing, users can now work together on metagenome binning, biosynthetic cluster and pathway study with colleagues. They can dynamically compare results using workspace profile functions or set operations to reach a group consensus and then resubmit the final datasets back into IMG, which can then be shared with community at large.

## Results and discussion

For many years IMG users have used the community annotation and user collaboration features in IMG for their research leading to information sharing and publications. We will describe three selected use cases in this section. None of the cases use all the provided features in IMG because certain features are irrelevant to their research and/or some new features were not available at their time of work (e.g., shared workspace was not available until June 2015). Therefore, we also present a mock scenario at the end of this section to show case how new IMG users can benefit the most.

### Xanthomonas study

Neha Potnis (University of Florida) and 11 colleagues have formed an IMG group for their *Xanthomonas* research. Three *Xanthomonas* genomes were submitted to IMG in 2009. The group used *Xanthomonas campestris pv. vesicatoria 85-10* as a reference for annotation and used the gene neighborhood feature to add MyIMG annotations. They also identified genes with incorrect starting codon, and those genes were marked obsolete. Finding missing gene function was used frequently to add genes that gene calling pipeline has failed to identify. They also found actual genes coded on opposite strands. The group commented that IMG platform allowed them to work collaboratively where scientists with expertise in different virulence systems could annotate the respective genes/clusters of their interest. After the three genomes have been properly annotated, new Genbank files were generated and submitted to NCBI. The new versions were added back into IMG in 2011:

- *Xanthomonas gardneri PDDCC 1620, ATCC 19865* (IMG Taxon OID: 651324109)
- *Xanthomonas perforans 91-118* (IMG Taxon OID: 651324110)
- *Xanthomonas vesicatoria Maraite*, ATCC 35937 (IMG Taxon OID: 651324111)

Their research lead to a publication [27], which is also listed in the genome detail page of the above three genomes.

#### Methanocella study

Zhe Lyu and advisor (China Agricultural University) have formed a group to study three *Methanocella* genomes. Their study focused on annotating genes identified by gene calling rather than adding missing genes. The users used gene neighborhood analysis to find operons, and used gene similarity search to find top homologs, orthologs and paralogs. Zhe also used other third party tools to build phylogenetic trees using results from IMG. The results from various methods assisted his MyIMG annotations. Zhe’s MyIMG annotations on the following two genomes were released to public and could be viewed by all IMG users:

- *Methanocella arvoryzae MRE50 (reannotation)* (IMG Taxon OID: 2505679073)
- *Methanocella paludicola SANAE (reannotation)* (IMG Taxon OID: 2505679075)

The research result was published [28, 29]. *Methanocella conradii HZ254* (IMG Taxon OID: 2512564055) was loaded into IMG in 2012, and the genome detail page lists [28] as genome publication.

#### Burkholderia study

Ann Hirsch (UCLA), colleagues and students have formed one of the largest IMG groups so far (more than 20 people). Their annotation effort has been running since 2009 and is still continuing. Because of the long involvement, this group also used the most community annotation and user collaboration features. Many IMG group features were inspired by their needs.

The group has used BLAST, homolog searches, and various comparative analysis tools provided by IMG to assist MyIMG annotations. Gene neighborhood analysis was used to check conservation of genes among different bacteria. Additional enzyme information was found using the finding missing enzyme function. The results aided their MyIMG annotations.

In addition to annotating existing genes, some genes were marked for removal from the genomes when there’s good evidence, mostly resulting from not finding similar genes using neighborhood searches. New genes were added using the finding missing gene function provided by IMG as well as by checking gene neighborhoods and using sequence similarity searches.

Their research results have been accepted for many publications including [30], which is listed as a genome publication of *Burkholderia tuberum STM678 (Burkholderia tuberum STM678T (IHQD assembly))* (IMG Taxon OID: 2512047030).

Recently the group also started experimenting with additional new features such as workspace in assisting group collaboration. There is also a plan to release MyIMG annotations upon the acceptance of their papers.

#### Mock scenario

We present here a mock scenario to show case how users can benefit the most from available IMG community annotation and user collaboration features.

A principal investigator (PI) first creates an IMG group to include all collaborators. Some collaborators can be assigned the role of co-owners to help with group administration. All relevant genomes and metagenomes for this research project can be saved in one or more workspace genome sets to be shared by all group members. PI can also use the grant genome permission feature in the IMG Group to grant access of private genomes and metagenomes to group members. A news item can be posted to inform members of the shared workspace genome sets to work on. A hyperlink to shared documents (e.g., Google Doc) can also be included in the news.

Group members then start researching on the genomes listed in the shared workspace genome datasets. They can save genes of interest to various workspace gene sets to be shared with other group members. Users can load contents of private or shared gene sets into Gene Cart and use a plethora of tools provided in IMG for analysis. They can add MyIMG gene annotations for gene product names, missing enzymes and additional protein information. They can also use sequence visualization tools and phylogenetic profiler to discover potential missing genes in an isolate genome. Gene neighborhood search results can be used to add additional annotations, to spot spurious genes, or to find new missing genes. Workspace gene sets and MyIMG annotations can be shared among group members so that users can compare results. Users can also view MyIMG gene annotations highlighted in a KEGG pathway map. If the research involves biosynthetic clusters and secondary metabolites (or natural products), then MyIMG SM annotations can also be added.

Once the research is complete, the PI can consolidate all MyIMG annotations. IMG provides a function for users to include MyIMG gene annotation to generate a Genbank file of an isolate genome. A user can then review and revise the Genbank file for new submission (e.g., to the IMG submission system). After the research result is accepted for publication, PI can make private genomes and corresponding MyIMG gene annotations public to be shared with community at large. New publications can be added to corresponding projects in the Genomes OnLine Database (GOLD) [31], and the publication information will be available to all IMG users from the genome detail pages.

## Conclusions

In this paper we present IMG features that support community annotation and user collaboration. IMG users can create IMG user groups to share genomes, user annotations and workspace datasets. They can also use various analysis and annotation tools in IMG to assist their research as described in detail in the implementation section. Case studies in the results section show that annotation can be part of the research process leading to knowledge sharing and academic publications.

We are able to address various issues encountered by other genomic annotation systems as follows:

- Usability: IMG provides integrated genomic information and various analysis tools to help users with their research and investigation. IMG users not only can add annotations to genes, but also can perform metagenome binning and form new gene clusters using tools provided by IMG, which is difficult to achieve using only a wiki-based system.
- Authorship recognition and tracing: IMG provides author recognition by linking annotations to users. Users can compare gene annotations of different authors in a list display, which is much easier and clearer than tracing through many versions of document editing. Publications (information obtained through GOLD) are listed in genome detail pages.
- User incentive: IMG annotation can be part of a research process as described in this paper. Users can incorporate their annotation results in the new version of genomes or simply release their existing private annotations upon the acceptance of their research paper. Since it requires minimal additional effort, we believe that users are more willing to participate.
- Reliability: Annotations are linked to real users. Genome detail pages list publications that have gone through strict peer review. Moreover, JGI experts are closely involved with public annotations, which greatly improves the reliability of the information. Help from JGI experts was acknowledged in many user publications.

Unlike most genomic annotation systems that only focus on one type of data (e.g., gene or pathway), IMG provides an integrated environment with genomes, genes, functions, pathways, etc. So far we have only provided user annotation features for genes (product name, protein information and enzyme), pathways (for experts only), biosynthetic clusters and secondary metabolites. There is no reason besides resource limitation that we cannot extend annotation capabilities to more gene features and/or to other types of objects in IMG. This will be an area for future improvement.

### Ethics

Not applicable.

### Consent to publish

Not applicable.

## Availability of data and material

IMG/ER (Expert Review) is available through URL http://img.jgi.doe.gov/mer. Browser requirements are described in the implementation section of this paper. IMG is committed to provide scientists worldwide free support for genome & metagenome data annotation & integration and open access comparative analysis of integrated genome and metagenomes. IMG users need to register at: JGI Single Sign On (JGI SSO) in order to obtain a login and password for gaining access to IMG’s data content and analysis tools (free of charge). Logins/passwords allow users to (i) submit their own genomes/metagenomes and keep them “private” for up to two years while they review and revise annotations; (ii) employ IMG’s curation tools for identifying and correcting annotation anomalies, such as protein products, for both private or public genomes-annotation revisions are recorded/saved in user specific “MyIMG” files on IMG’s file system; (iii) employ IMG’s Workspace which supports a persistent version of IMG’s “Carts” and performing long running analysis computations; (iv) download IMG genome and metagenome datasets via JGI’s Portals.

## Abbreviations

BC: biosynthetic cluster
COG: Clusters of Orthologous Groups
IMG: Integrated Microbial Genomes system
JGI: DOE Joint Genome Institute
KEGG: Kyoto Encyclopedia of Genes and Genomes
KO: KEGG orthology
NP: natural product
SM: secondary metabolite
